# Challenges in the Extracorporeal Membrane Oxygenation Era

**DOI:** 10.3390/membranes11110829

**Published:** 2021-10-27

**Authors:** Marco Giani, Antonio Arcadipane, Gennaro Martucci

**Affiliations:** 1 ASST Monza, Emergency and Intensive Care, 20900 Monza, Italy; marco.giani@unimib.it; 2 School of Medicine and Surgery, University of Milano-Bicocca, 20900 Monza, Italy; 3 Department of Anesthesia and Intensive Care, IRCCS-ISMETT, UPMC Italy, 90133 Palermo, Italy; aarcadipane@ismett.edu

In the last decade, the use of extracorporeal membrane oxygenation (ECMO) has significantly increased. Thanks to technological advances, such as the improved biocompatibility of extracorporeal surfaces, together with recent improvements in clinical management, ECMO now plays a pivotal role to support patients with severe respiratory failure. In fact, its use has expanded to bridge to lung and cardiac transplantation, as well as to ventricular assist devices, and it is being increasingly used year after year, its potential contraindications trailing behind the successful expansion of its applications.

The *Membranes* Special Issue “Challenges in the new Extracorporeal Membrane Oxygenation Era” sought contributions to explore the current borders of ECMO applications. This Special Issue is a large summary of some debated topics for which definitive evidence is lacking, with the right mixing of original articles and sharp review. Moreover, in this Special Issue, a global view was actively prompted, and the contribution from centers distributed in different countries and continents gives the opportunity for sharing knowledge and standardization of practice.

The first debated topic is the adequate patient selection for ECMO, at a time when the indications for ECMO are broadening. As an historical overview, with the aim to indicate the future pathway, Feldhaus and colleagues describe [1] the steps moved forward by ECMO as respiratory support. Giani and colleagues sum up the open questions, the controversies and the future directions of extracorporeal gas exchange in the context of acute respiratory distress syndrome (ARDS) [2]. Specifically, they discussed the current evidence and the debated aspect of sedation, patient and renal replacement therapy [3], anticoagulation during ECMO, and prone positioning [4] during extracorporeal support. Following this pathway, a thorough evaluation of contraindications to extracorporeal gas exchange [5] (reducing year by year), the principles of prognostication during ECMO [6] and the use of ECMO in thoracic surgery have been explored [7]. An interesting review by Marchiori et al. reviewed the current scientific literature about ECMO donors, focusing on the use of ECMO tissues as allografts [8]—a topic that will be probably become relevant in the next years. As an ideal conclusion of this chapter, two original studies focused on outcomes of ARDS patients supported by ECMO; Chiu [9] showed that propensity score-matched ARDS patients treated with ECMO are more likely to survive than patients on conventional protective mechanical ventilation, and this benefit seemed greater for the most severe patients and when the extracorporeal support was initiated early (i.e., within 48 h); Martucci and colleagues [10] defined a set of microRNAs which may provide new insights on the processes involved in the pathogenesis and evolution of ARDS and may represent promising biomarkers to evaluate ongoing treatments and for prognostication.

In the last two years, the COVID-19 pandemic brought a dramatic increase in ECMO use. In this Special Issue, this topic is dealt with from different perspectives. As described in the papers by Montrucchio et al. [11] and Dave et al. [12], COVID-19 forced a reorganization of the intensive care departments, which involved care teams, spaces and required adequate planning.

Furthermore, as discussed by Shah and colleagues [13], intensivists had to face new clinical challenges. The shortage of mechanical ventilators pushed clinicians to investigate the possibility of sharing a ventilator, which raised a number of physiological questions such as circuit cross-flows and patient interactions, as discussed by Colombo et al. [14]. In order to minimize the exposure of health care personnel, even routine clinical procedures such as percutaneous tracheostomy were modified [15]. Immunomodulation raised as a cornerstone therapy for COVID-19-associated ARDS, and therapies such as steroids and Tocilizumab were introduced and scientifically evaluated [16].

COVID-19 often showed as a pulmonary disease, but the underlying vasculopathy often led to a myocardial or coagulative involvement, which required a change from veno-venous ECMO to other configurations in up to 18% of cases [17]. Finding the balance between hypercoagulable state and bleeding risk was a major challenge, as discussed in the case report by Khalil and colleagues [18].

The need to improve simulation and training in the ECMO field was also investigated in this article series, which includes the description [19] of development of a modular ECMO simulator to enhance the training process, and the development of an advanced thermochromic ink system for medical blood simulation [20].

Experimental studies shed a new light on the ECMO future. The aim of minimizing the mechanical ventilation burden on the sick lung was explored in a porcine model, where extracorporeal gas exchange allowed “ultralow” tidal volume ventilation to be achieved [21]. Alternatives to the fresh gas flow were investigated: Vivona et al. [22] demonstrated the efficacy and feasibility of alkaline liquid ventilation, which was capable in achieving a CO_2_ removal capacity comparable to 10 L/min of oxygen. Recent technical development aimed at improving ECMO biocompatibility, reducing the need for transfusions [23] and, ultimately, improving outcomes. Causative factors of in-vitro hemolysis during ECMO were explored in a study [24] by Chan and colleagues. The level of hemolysis was higher in male donors, in heparinized blood (compared to citrated blood), and with lower blood flow rates (1.5 vs. 4 L/min). Trends, advantages and disadvantages in anticoagulation and coating methods used in extracorporeal life support devices were discussed [25] by Willers et al.

Some articles also tackle the specific debate of ECMO management. Hildreth and colleagues provided a new vision of ECMO retrieval [26], which may extend beyond the limits of national borders, highlighting how centralization of patients is fundamental in modern ECMO practice. Hughes and colleagues gathered the evidence on packed red blood cell transfusion during ECMO [27]. Last, the pathophysiology of left ventricle distention and the strategies of left ventricle decompression in patients supported with venoarterial ECMO were reviewed [28] by Ricarte Bratti et al.

Several aspects of ECMO setup, management, and characteristics are still obscure, and knowledge of this topic needs continuous updating. In this light, “ECMO-logy” has become a distinct discipline with a vast and peculiar background. With this Special Issue, we took a step forward in exploring this evolving discipline.

## Data Availability

Not applicable.
